# Molecular Profiling of 22 Primary Atypical Meningiomas Shows the Prognostic Significance of 18q Heterozygous Loss and *CDKN2A/B* Homozygous Deletion on Recurrence-Free Survival

**DOI:** 10.3390/cancers13040903

**Published:** 2021-02-21

**Authors:** Valeria Barresi, Michele Simbolo, Adele Fioravanzo, Maria Liliana Piredda, Maria Caffo, Claudio Ghimenton, Giampietro Pinna, Michele Longhi, Antonio Nicolato, Aldo Scarpa

**Affiliations:** 1 Department of Diagnostics and Public Health, Section of Pathology, University of Verona, 37134 Verona, Italy; simbolo.michele@hotmail.it (M.S.); lilianapiredda@yahoo.it (M.L.P.); aldo.scarpa@univr.it (A.S.); 2 Unit of Anatomic Pathology, S. Bortolo Hospital, 36100 Vicenza, Italy; adele.fioravanzo@aulss8.veneto.it; 3 Department of Biomedical and Dental Sciences and Morphofunctional Imaging, Section of Neurosurgery, University of Messina, 98125 Messina, Italy; mcaffo@unime.it; 4 Unit of Pathology, Department of Pathology and Diagnostics, University and Hospital Trust of Verona, 37126 Verona, Italy; claudio.ghimenton@aovr.veneto.it; 5 Unit of Neurosurgery, Department of Neurosciences, Hospital Trust of Verona, 37126 Verona, Italy; giampietro.pinna@aovr.veneto.it; 6 Unit of Stereotaxic Neurosurgery, Department of Neurosciences, Hospital Trust of Verona, 37134 Verona City, Italy; michele.longhi@aovr.veneto.it (M.L.); antonio.nicolato@aovr.veneto.it (A.N.); 7 ARC-Net Research Centre, University and Hospital Trust of Verona, 37134 Verona, Italy

**Keywords:** atypical meningioma, tumor mutational burden, recurrence, *NF2*, *CDKN2A*/*B*, *AKT1*, 18q, brain invasion, *MSH2*, microsatellite instability

## Abstract

**Simple Summary:**

The post-surgical treatment of atypical meningiomas is controversial in cases with gross total resection, because one out of two patients develops disease recurrence at five years. Factors able to predict the risk of recurrence may be useful in selecting patients who require adjuvant treatment. In this study, we analyzed the molecular alterations of 22 atypical meningiomas undergoing complete surgical resection and their statistical correlation with the risk of recurrence. The loss of one copy of chromosome 18q and the loss of both copies of *CDKN2A/B* gene were significantly associated with a shorter time to recurrence. Therefore, we suggest that atypical meningiomas could be tested routinely for these genetic alterations to identify cases for adjuvant treatment.

**Abstract:**

The use of adjuvant therapy is controversial in atypical meningiomas with gross total resection. Predictors of recurrence risk could be useful in selecting patients for additional treatments. The aim of this study was to investigate whether molecular features are associated with recurrence risk of atypical meningiomas. According to WHO classification, the diagnosis of atypical meningioma was based on the presence of one major criteria (mitotic activity, brain invasion) or three or more minor criteria. The molecular profile of 22 cases (eight mitotically active, eight brain-invasive, and six with minor criteria) was assessed exploring the mutational status and copy number variation of 409 genes using next generation sequencing. Of the 22 patients with a median follow up of 53.5 months, 13 had recurrence of disease within 68 months. *NF2* mutation was the only recurrent alteration (11/22) and was unrelated to clinical-pathological features. Recurring meningiomas featured a significantly higher proportion of copy number losses than non-recurring ones (*p* = 0.027). Chromosome 18q heterozygous loss or *CDKN2A/B* homozygous deletion was significantly associated with shorter recurrence-free survival (*p* = 0.008; hazard ratio: 5.3). Atypical meningiomas could be tested routinely for these genetic alterations to identify cases for adjuvant treatment.

## 1. Introduction

Meningiomas are the most frequent tumors of the central nervous system [1]. They are currently classified by the World Health Organization (WHO) into three grades of malignancy, with each grade having several histological variants [1].

The WHO histological grade and the extent of surgery, classified according to the Simpson resection grading scale [2,3], represent the most relevant prognostic factors in patients affected by these neoplasms [2]. Because recurrence rate progressively increases with the histological grade (7–25% for grade I, 29–52% for grade II, and 50–94% for grade III) [1], patients with grade I meningioma do not receive any additional treatment after gross total resection, while those with grade III meningioma are invariably sent to adjuvant therapies [4]. The post-surgical treatment of grade II meningioma is still controversial in cases with gross total resection; indeed, observation and fractioned radiotherapy are both considered as options [4,5,6].

Among histological grade II meningiomas, the atypical histotype is the most frequent. WHO diagnostic criteria for this variant are: (i) mitotic index of 4–19 mitoses per ten high power fields (HPFs); or (ii) brain invasion; or (iii) at least three minor criteria including spontaneous necrosis, pattern-less architecture (sheeting), small cells with high nuclear/cytoplasmic ratio, macronucleoli, and hypercellularity [1]. Therefore, atypical meningiomas are a heterogeneous group, which encompasses some tumors featuring clear biological aggressiveness, documented by a high mitotic count or infiltrative growth pattern, and others with less obvious malignant potential and featuring only minor atypical criteria. A recent multicentric study on 200 cases showed that atypical meningiomas featuring brain invasion or only minor atypical criteria are less prone to recurrence than those with high mitotic counts [7]. Moreover, a score based on the presence of at least two high-risk features, among male sex, sagittal site, Simpson grade 3, sheeting, and a mitotic index ≥ 6/10 HPFs, was found to be a significant and independent predictor of recurrence in patients with atypical meningiomas [7]. 

The most frequent cytogenetic alteration in meningiomas is the loss of chromosome 22q [8,9]. This latter harbors the *NF2* gene, which is inactivated by copy number variations and/or point mutations in around 60% of cases [9,10,11,12,13]. Tumors with intact *NF2* have mutations in other genes, including *AKT1*, *SMO*, *TRAF7*, and *KLF4* [14,15]. 

The mutational spectrum of atypical meningiomas is mainly characterized by *NF2* mutations [16] that co-occur with genomic instability or recurrent *SMARCB1* mutations in primary atypical meningiomas, and with *TERT* promoter mutations in secondary (i.e., arisen from the progression of benign tumors) ones [17]. 

A recent study showed that grade II meningiomas are molecularly heterogeneous and, based on the mutational status of *NF2*, *TERT* promoter, *TP53*, *BAP1*, *PRBM1*, *AKT1*, *SMO* and *PIK3CA,* they can be subgrouped into three different categories, each characterized by peculiar clinical-pathological features [9]. Other studies demonstrated that the integration of the histopathological diagnosis with the methylation profile, karyotype, or mutational status of the *TERT* promoter could allow for a better prognostic stratification of meningiomas [18,19,20,21,22]. Nonetheless, whether the genetic and histological characteristics of atypical meningiomas are related to each other remains to be clarified.

In this study, we aimed to analyze whether the molecular alterations of atypical meningiomas are correlated with their histopathological features or recurrence risk.

## 2. Materials and Methods

### 2.1. Cases

We selected 22 primary atypical meningiomas, recurring or not, including 8 cases with a high mitotic index (≥6 mitoses per 10 HPF) (Group 1), 8 featuring brain invasion (Group 2), and 6 with only minor criteria (three or more, including sheeting and spontaneous necrosis) (Group 3).

### 2.2. Ethical issues

This study was approved by Ethics Committee of for Clinical Experimentation of Verona and Rovigo Provinces Comitato Etico per la Sperimentazione Clinica delle province di Verona e Rovigo (protocol n. 40400, 2019/07/19).

### 2.3. Clinical Data

Information on the localization, extent of surgical resection, and development of recurrences was retrieved using operatory registries and clinical records.

The extent of surgical resection was graded according to Simpson scale [2], into: grade 1 (complete excision, including dura and bone); grade 2 (complete excision plus apparently reliable coagulation of dural attachments); and grade 3 (complete excision of the solid tumor, but insufficient dural coagulation or bone excision). 

Recurrence was defined as the identification of a tumor in the site of previous surgery by means of computerized tomography or magnetic resonance imaging. Recurrence-free survival (RFS) was the length of survival to the detection of a recurrent tumor.

### 2.4. Mutational and Copy Number Variation Status of 409 Cancer Genes

DNA was obtained from 10 formalin-fixed paraffin embedded (FFPE) consecutive 4 μm sections using the QIAamp DNA FFPE Tissue Kit (Qiagen, Milan, Italy) and qualified as previously reported [23]. The targeted next-generation sequencing (NGS) panel used was the Oncomine Tumor Mutational Load (TML) assay (ThermoFisher), which covers 1.65 Mb of genomic space for the assessment of tumor mutational burden and explores all exons of 409 cancer-related genes for mutational and copy number assessment.

Sequencing was performed on Ion Torrent platform using 20 ng of DNA for each multiplex PCR amplification and subsequent library construction. The quality of libraries was evaluated using the Agilent 2100 Bioanalyzer on-chip electrophoresis (Agilent Technologies). Libraries were clonally amplified by emulsion PCR with Ion OneTouch OT2 System (Thermofisher) and sequencing was run on Ion Proton (Thermofisher) loaded with Ion PI Chip v3.

Torrent Suite Software v.5.10 (Thermofisher) was used for data analysis, including alignment to the hg19 human reference genome and variant calling. Filtered variants were annotated using a custom pipeline based on vcflib (https://github.com/ekg/vcflib (accessed on 10 May 2020)), SnpSift [24], Variant Effect Predictor (VEP) [25], and the NCBI RefSeq database. Additionally, alignments were visually verified with the Integrative Genomics Viewer (IGV) v2.3 [26] to confirm the presence of identified mutations. Copy number variation (CNV) was evaluated using OncoCNV v6.8 [27], comparing the BAM files obtained from tumor samples with those obtained from blood samples of four healthy male subjects. 

The software includes a multi-factor normalization and annotation technique, enabling the detection of large copy number changes from amplicon sequencing data and permits to visualize the output per chromosome. 

### 2.5. Tumor Mutational Burden

Tumor mutational burden (TMB) and mutational spectrum were evaluated using the Oncomine TML 5.10 plugin available on IonReporter software (Thermofisher, Milan, Italy). Default modified parameters were used to exclude sequencing artefacts. In detail, a threshold of at least 20 reads and an allelic frequency of 10% of variant was used for mutation calling. TMB is expressed as the number of mutations per Mb (muts/Mb), where mutations include nonsynonymous missense and nonsense single nucleotide variants (SNVs), plus insertion and deletion variants (InDels) detected per Mb of exonic sequences.

### 2.6. Immunohistochemistry of DNA Mismatch Repair Proteins

Immunostaining was performed using the Bond Polymer Refine Detection kit (Leica Biosystems) in a BOND-MAX system (Leica Biosystem, Milan, Italys) on 4 μm-thick FFPE sections using the following primary antibodies purchased from Leyca Biosystems: mouse monoclonal clones ES05 against MLH1 (dilution 1:30), 79H11 against MSH2 (dilution 1:30), PU29 against MSH6 (dilution 1:100) and MOR4G against PMS2 (dilution 1:100). Normal cells within the samples acted as positive internal controls.

### 2.7. Microsatellite Instability Analysis

Microsatellite Instability (MSI) was tested by a fluorescent multiplex PCR exploiting the 5 mononucleotide microsatellites BAT25, BAT26, NR21, NR22, NR24. Amplicons were separated by capillary electrophoresis using the ABI Genetic Analyzer 3130XL (Applied Biosystems). Variations ≥3bp for BAT25, NR21, NR22, NR24 and ≥4bp for BAT26 were considered as instability.

### 2.8. Statistical Analyses

Statistical difference in TMB or in copy number losses among clinical-pathological groups was explored using the Kruskal–Wallis test. 

The statistical correlations between chromosomal deletions and clinical-pathological parameters were analyzed by the chi-squared test, as were the correlations between genetic, clinical, or pathological features and recurrence. 

Finally, RFS was assessed by the Kaplan–Meier method, with the date of primary surgery as the entry data and length of survival to the detection of a recurrent tumor as the end point. The Mantel–Cox log-rank test was applied to assess the strength of association between RFS and each of the parameters as a single variable. 

A probability (*p*) value less than 0.05 was considered significant. Statistical analysis was performed using MedCalc 12.1.4.0 statistical software (MedCalc Software, Mariakerke, Belgium). 

## 3. Results

### 3.1. Cases

**Figure 1:**
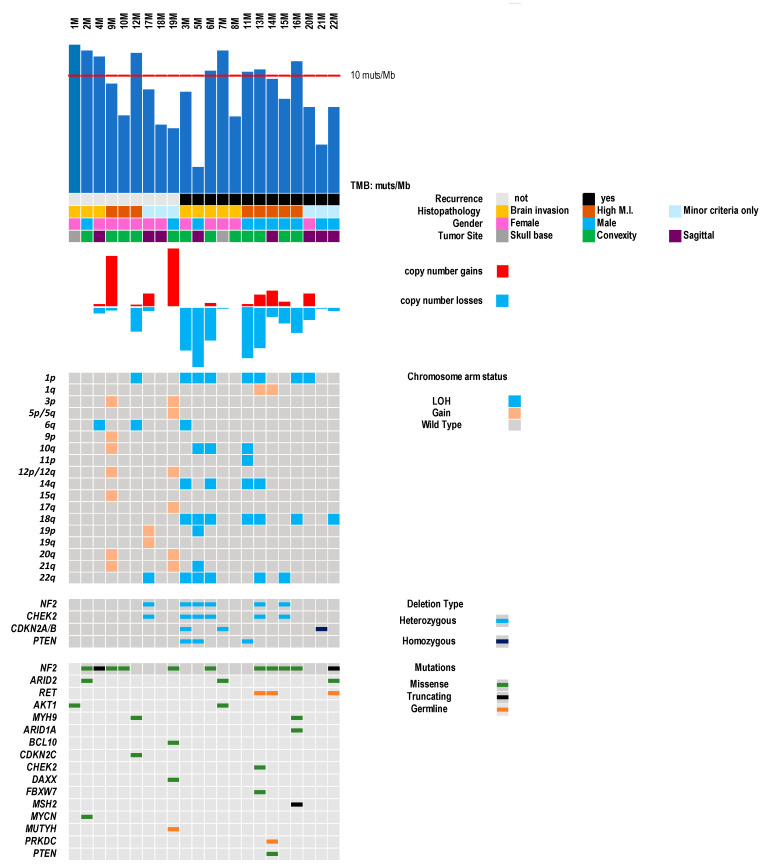
Genomic landscape of 22 atypical meningiomas. The matrix shows 18 genes that were altered at sequencing analysis, and the main chromosomal alterations. Samples are ordered according to the presence of recurrence and meningioma histopathological group.

Twelve meningiomas were from female patients and 10 were from male patients (age range: 36–83 years; median: 68 years) (Table 1 and Figure 1).

All meningiomas had been totally removed and were Simpson grade 1 in 13 cases, grade 2 in 5, and grade 3 in 4. No patients received adjuvant radiotherapy.

RFS ranged between 47 and 107 months in patients without recurrence (median: 72 months), and between 4 and 68 months in those who developed recurrences (median: 12 months).

### 3.2. Mutational and Copy Number Variation Status of 409 Cancer Genes

A total of 32 mutations in the coding regions of 16 genes were identified (Table S1).

Fourteen of the 22 cases had at least one gene mutation, while eight cases (3M, 5M, 8M, 11M, 17M, 18M, 20M, 21M) had no mutations in any of the 409 genes analyzed, but only variations in untranslated genomic regions (Table S1; Figure 1). The CNV status was estimated for all 409 genes using sequencing data. 

Fourteen meningiomas (63%) had *NF2* alterations, consisting of: (i) *NF2* mutations coupled with the deletion of the second allele in three cases (6M; 13M and 15M); (ii) monoallelic *NF2* mutations in eight cases (2M, 4M, 9M, 10M, 14M, 16M, 18M and 22M); (iii) *NF2* heterozygous deletion in three cases (3M, 5M, 17M) (Figure 1). *NF2* alterations were not significantly associated with any clinical-pathological features or meningioma histopathological group.

Three meningiomas, all recurring within 68 months of follow-up, had *RET* alterations, consisting of an R982C mutation in two cases (13M; 22M) and a Y791F mutation in one case (14M). Two brain invasive meningiomas, both localized at the anterior medial skull base (olfactory groove), had alterations in the *PI3K-AKT-mTOR* pathway, consisting of *AKT1* E17K mutations (1M; 7M), which were mutually exclusive to *NF2* alterations. 

Two recurring meningiomas had the inactivation of cell cycle inhibitors; one (13M) had a *CHEK2* truncating mutation, coupled with the deletion of the second allele, the other (21M) had *CDKN2A/B* homozygous deletion. Finally, one meningioma (16M) had an *MSH2* truncating mutation. 

We did not find any statistical association between gene alterations and clinical-pathological features. 

### 3.3. Chromosomal Alterations

Based on the chromosomal position of each gene, the status of chromosome arms was inferred. The most frequent chromosomal alteration was 1p (eight cases) deletion, followed by 18q (seven cases), 22q (six cases), 14q (four cases), 6q (three cases) and 10q (three cases) heterozygous deletions (Figure 1).

The presence of 18q heterozygous deletion was significantly associated with the development of tumor recurrence (*p* = 0.009). Although statistical significance was not reached, the deletion of 1p (*p* = 0.11), 10q (*p* = 0.12), 14q (*p* = 0.07), 22q (*p* = 0.166) was more frequent in atypical meningiomas that recurred than in non-recurring ones.

No significant association between chromosomal alterations and other clinical-pathological features was found. 

For each case, the total copy number losses (CNL) and gains (CNG) were calculated. CNL ranged between 0 and 74 (median: 8.5; mean: 22); CNG ranged between 0 and 86 (median: 1; mean: 13.5). Meningiomas that recurred had a significantly higher count of CNL than those which did not recur (*p* = 0.027).

### 3.4. Tumor Mutational Burden

The number of mutations/Mb in the 22 atypical meningiomas ranged between 2.1 and 12.6 (median: 9; mean: 8.7). Nine cases (five in Group 2 and four in Group 3) had ≥10 mutations/Mb (Figure 1; Table 1). 

Meningiomas in groups 1 and 2 had significantly higher TMB than meningiomas in Group 3 (Groups 1–2, median: 10.3, IQ: 8.3–11.7; Group 3, median: 6.5, IQ: 5.5–7.3) (*p* = 0.012). In addition, meningiomas at the skull base had significantly higher TMB (median 12.4, IQ: 12.1–12.6) than meningiomas at the convexity (median: 9.7, IQ: 7.3–11) or sagittal meningiomas (median: 7.3, IQ: 4.9–9.3) (*p* = 0.033). 

The meningioma with *MSH2* mutation (16M) had a TMB of 11.42 muts/Mb.

### 3.5. Immunohistochemistry of DNA Mismatch Repair Proteins and Microsatellite Instability Analysis

**Figure 2:**
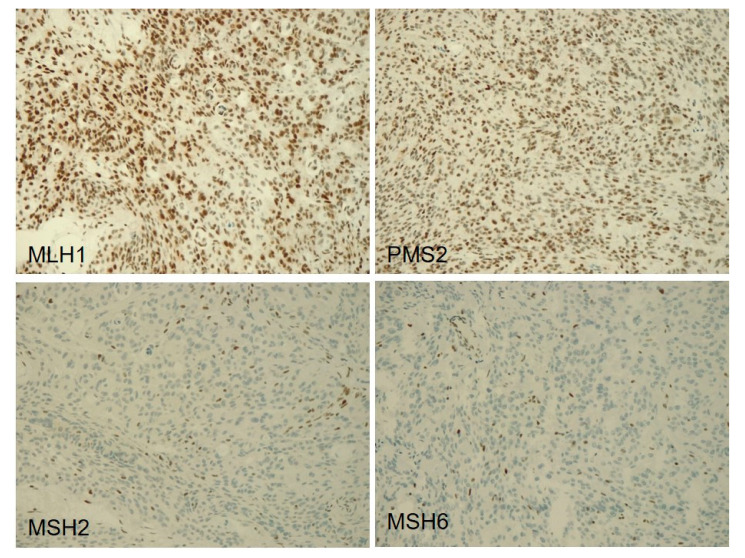
Immunohistochemistry for mismatch repair (MMR) proteins in a meningioma with *MSH2* mutation. Nuclear expression of MLH1 and PMS2 was retained in the neoplastic cells of meningioma, while that of MSH2 and MSH6 was lost in the neoplastic cells and retained in non-neoplastic cells (original magnification, ×200).

One atypical meningioma (16M), carrying an *MSH2* truncating mutation, had loss of MSH2 and MSH6 immunostaining in all tumor cells (Figure 2), but none of the five microsatellites analyzed were unstable.

All the other meningiomas retained the expression of mismatch repair proteins and the microsatellites were stable. 

### 3.6. Recurrence-free Survival Analyses

For each case, we calculated a clinical-pathological risk score, by assigning 1 point to the presence and 0 to the absence of each parameter including male sex, sagittal site, sheeting, mitotic index ≥10 HPF, and Simpson grade 3, as previously suggested [7]. A score ≥ 2 was considered to be high risk [7].

Univariate analyses showed that a high clinical-pathological risk score (hazard ratio: 3.5; 95% confidence interval: 1.1–11.1; *p=* 0.03) and 18q heterozygous deletion (hazard ratio: 3.8; confidence interval: 1.1–13.8; *p* = 0.03) were significantly associated with a shorter RFS (Table 2).

**Figure 3:**
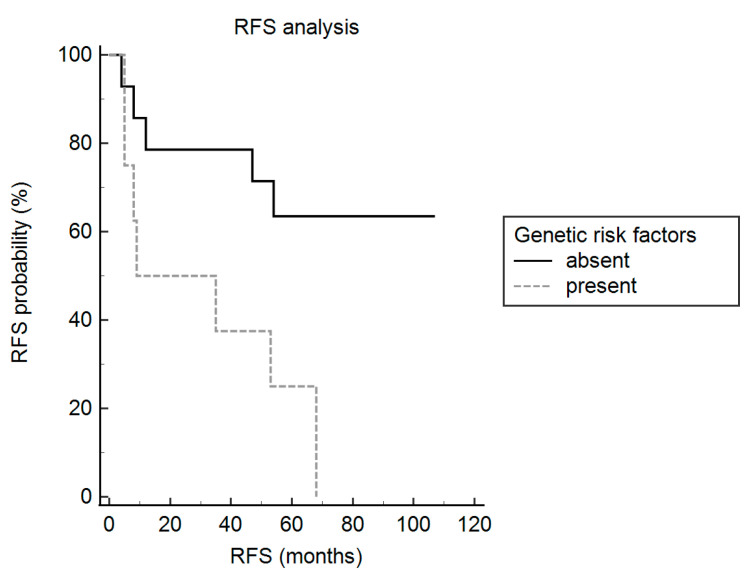
Impact of genetic risk factors on clinical outcome. RFS analysis showed that the presence of 18q heterozygous deletion or *CDKN2A/B* homozygous deletion is associated with worse prognosis in atypical meningiomas (*p* = 0.008).

Then, cases were classified into: (i) high genetic risk, when having 18q heterozygous deletion or *CDKN2A/B* homozygous deletion; (ii) low genetic risk, when those genetic features were absent. High genetic risk was significantly associated with the development of recurrence (*p* = 0.0039) and with shorter RFS (hazard ratio: 5.3; confidence interval: 1.5–18.2; *p* = 0.008) (Table 2 and Figure 3), as well.

To validate our findings in larger cohorts including meningiomas of all WHO grades, we extrapolated the raw data from the study by Abedalthagafi et al. [28]. One hundred and eight cases were primary tumors with available data on time to recurrence. They included 85 WHO grade I, 22 WHO grade II, and 1 WHO grade III meningiomas. One case had the loss of one or two copies of *CDKN2A/B*, 11 meningiomas had the loss of 18q, while these genetic alterations co-occurred in 2 cases. Therefore, a total of 12 meningiomas (4 WHO grade I; 7 WHO grade II; 1 WHO grade III) had a high genetic risk. The Mantel–Cox log-rank test showed that patients harboring meningiomas with a high genetic risk had a significantly shorter RFS than those having meningiomas with a low genetic risk (hazard ratio: 4.8; confidence interval: 0.7–33.7; *p* = 0.005). 

Since the presence of necrosis was associated with a bad outcome in atypical meningiomas [29], we investigated its prognostic relevance in this cohort. Although meningiomas featuring spontaneous necrosis had shorter RFS, statistical significance was not reached (hazard ratio: 2.7; confidence interval: 0.8–8.5; *p* = 0.087).

## 4. Discussion

The post-surgical treatment of totally resected atypical meningiomas is still a matter of debate, because 50% of the patients develop disease recurrence within five years from the initial diagnosis. In this perspective, the identification of clinical, histological, or genetic factors associated with a higher risk of recurrence may be helpful to select patients who could benefit from adjuvant therapies. In addition, the definition of their molecular profiling may contribute to finding novel treatments for atypical meningiomas. 

In this study, we assessed the molecular profile of 22 primary atypical meningiomas with gross total resection, exploring the mutational status and copy number variation of 409 genes, and TMB as well.

We did not find any recurrent gene mutations, with the exception of that of *NF2*, which was present in 50% of cases. This is in accordance with other studies reporting *NF2* as the only gene frequently mutated in atypical meningiomas [13,16,17]. Of note, the *NF2* mutation did not associate with the histological group or with clinical features. 

Among the *NF2-*mutated meningiomas, two cases, resected from male patients and located at the convexity, showed 1p and/or 22q deletion, and *ARID1A* or *FBXW7* mutation. These cases could follow the canonical *NF2*-mutant pattern of progressive meningioma recently described by Williams et al. [9]. This, indeed, is characterized by mutations of several genes including *ARID1A* and *FBXW7,* by 1p and/or 22q deletion, and by the association with male sex and localization at the convexity, in addition to *NF2* mutation [9]. Two *NF2* unaltered meningiomas, localized at the olfactory groove and brain-invasive, had *AKT1* E17K mutation. This confirms the association between *AKT1* mutation and meningioma localization at the anterior or middle skull base [14,15,28] and suggests that this genetic alteration might be correlated with brain invasion in the atypical histotype. Indeed, *AKT1* mutation was previously found in two out of nine brain-invasive meningiomas [30] and in only 1/81 atypical meningiomas [14,15,28,31] classified according to WHO 2007, which do not include brain invasion among the diagnostic criteria [1]. 

Although initially believed to be exclusive to grade I meningiomas, *AKT1* mutation was found in 2% of grade II meningiomas in another recent study [9]. Progressive meningiomas harboring this mutation have been categorized in the *NF2*-exclusive pattern, that lacks *NF2* mutation and associates with skull base localization and female sex [9], similarly to the two *AKT1*-mutated atypical meningiomas identified in this study. 

Three atypical meningiomas were *RET* mutated. *RET* R982C and Y791F mutations were reported in patients with multiple endocrine neoplasia type 2 [32,33,34], but unreported in meningiomas up to now. Although initially believed to be pathogenic for thyroid cancer, these mutations were recently demonstrated to have a pathogenic effect only if co-occurring with other *RET* mutations [32,33,34]. 

One atypical meningioma had a pathogenic truncating mutation of *MSH2,* associated with the immunohistochemical loss of the corresponding protein and of its secondary partner MSH6. Inactivating mutations in *MMR* genes were previously reported in 3 out of 1081 meningiomas [35]. Similarly to the *MSH2* mutated meningioma in this cohort, all three cases were hypermutated (i.e., had a TMB of > 10 mutations/Mb according to Campbell et al. [36]), suggesting that mismatch repair (MMR) deficiency is associated with hypermutation in meningiomas, as in other solid tumors [37,38]. 

Of note, an anecdotal report showed that hypermutated meningiomas with MMR deficiency may respond to immune check-point blockade [35]. The formal proof that the MMR machinery is not functioning is the detection of MSI by molecular assays testing mononucleotide microsatellites; however, immunohistochemistry to detect the loss of MMR proteins is routinely used as a surrogate [39]. The absence of MSI in the *MSH2* mutated meningioma of our series indicates that, in meningiomas, the immunohistochemical loss of MMR proteins is not a good alternative to MSI testing and should not be used to select patients for treatment with immune check-point inhibitors, as already suggested in gliomas [40]. Of note, MSI was found in only one of 771 progressive meningiomas in a recent study, showing that this is a rare event in these tumors [9].

The few studies assessing TMB in meningiomas reported a low frequency of hypermutation [9,16,35]. Indeed, 9/850 cases had > 20 mutations/Mb in Williams et al. [9], and 21/1080 had > 10 mutations in Dunn et al. [35]. In this latter study, only six hypermutated meningiomas had mutations in genes of DNA repair systems, while the mechanism underlying hypermutation remained unclarified in the remaining 15 [35]. Similarly, the nine hypermutated meningiomas in this cohort had no recurring mutations; however, cases featuring a high mitotic index, or brain invasion, or localization at the skull base, had a significantly higher TMB. 

In accordance with the higher frequency of copy number variation burden reported in grade II/III meningiomas compared to grade I ones, atypical meningiomas of this series had a high proportion of copy number alterations [17,22,41]. Notably, the cases that recurred had a significantly higher count of CNL than those which did not recur. These latter instead mainly featured CNG. 

*NF2* heterozygous deletion was the most frequent (6/22 cases) CNL and it was invariably associated with the heterozygous deletion of *CHEK2.* This is a tumor suppressor gene, involved in DNA repair, and its loss has been associated with genomic instability and progression in meningiomas [42]. In accordance with this, in our series, the meningioma with the bi-allelic inactivation of *CHEK2* had multiple chromosomal losses and CNL, and it recurred during the follow-up. 

One meningioma that featured only minor atypical criteria had the homozygous deletion of *CDKN2A/B*, in the absence of *NF2* mutation. It followed an aggressive clinical course, and recurred in five months, in spite of gross total resection. Such an adverse outcome is in line with the reported association between *CDKN2A/B* homozygous deletion and higher recurrence risk of meningiomas [21,43,44]. In a series of 30 cases of variable histotype and grade, the homozygous deletion of *CDKN2A/B* was identified in three anaplastic (grade III) meningiomas with ≥ 20 mitoses/10 HPF and that recurred [43]. Our findings show that *CDKN2A/B* homozygous deletion can be also found in atypical meningiomas, in the absence of a high mitotic index. Progressive meningiomas featuring *CDKN2A/B* homozygous deletion and lacking *NF2* mutations were recently categorized in the *NF2* agnostic pattern, which is characterized by mutations in *TP53* or *TERT* promoter [9]. Although the *CDKN2A/B* deleted meningioma in this study had no *TP53* mutations, we did not assess the mutational status of *TERT* promoter to confirm its possible categorization in the *NF2* agnostic pattern.

CNL in meningiomas of this cohort were mainly clustered at some chromosomal segments, namely 1p, 6q, 10q, 14q, 18q, and 22q. In accordance with this, the loss of one copy of these chromosomes was previously associated with histological grades II and III of meningiomas [17,20,22,30,41,45].

Based on the findings in a series of 24 cases, McNulty et al. concluded that the loss of chromosome 10 could be used as a diagnostic marker of grade III meningioma [30]. However, we showed that this chromosomal alteration can be also present in grade II meningiomas, both mitotically active or brain invasive. 

With the exception of 6q, all the other chromosomal deletions were more frequent in recurring meningiomas; notably, the heterozygous deletion of 18q was significantly associated with the development of recurrence and with a shorter RFS. The association between single copy loss of 1q, 6q, 14q, or 18q and the recurrence of meningiomas was already demonstrated [18,20]. In addition, Patel et al. identified one group of meningiomas (which they named group C) that was characterized by shorter time to recurrence and that was mainly composed of grade II and III tumors with frequent losses in chromosomes 1p, 3p, 4p, 6q, 14p, 14q, 18q, and 22q [45]. However, this is the first report showing the significant prognostic value of 18q heterozygous deletion in the subgroup of atypical meningiomas. In this cohort, patients harboring tumors with 18q heterozygous deletion or *CDKN2A/B* homozygous deletion had significantly shorter RFS. In addition, the hazard ratio associated with the presence of these genetic alterations was greater than that of a high clinical-pathological risk score, based on the presence of at least two high-risk features among male sex, sagittal site, Simpson grade 3, sheeting, and a mitotic index ≥ 6/10 HPFs [7]. 

Aizer et al. previously formulated a so-called cytogenetic abnormality score, based on *CDKN2A/B* homozygous loss, single copy loss of 1p, 4p, 6q, 7p, 10q, 11p, 14, 18q, or 19q, and monosomy of 22 [22]. They showed that a high score, dichotomized for its median value (3.5), was significantly associated with lower RFS in a cohort of 32 atypical meningiomas treated with gross total resection [22]. However, the risk score based on two genetic variables, which we propose in this study, might be used more easily in routine practice.

## 5. Conclusions

The diverse histopathological groups of atypical meningiomas (mitotically active, brain-invasive, or with only minor atypical criteria) do not have significantly different genetic features. However, tumors with a high mitotic index or brain invasion have significantly higher TMB than those with only minor atypical criteria. In addition, *AKT1* mutations seem to be preferentially associated with brain invasion. Albeit rarely, atypical meningiomas may show *MMR* mutations, but these may not lead to MSI. 

Finally, even in the absence of a high mitotic index or brain infiltration, atypical meningiomas may feature *CDKN2A/B* homozygous deletion. This and 18q heterozygous loss are significant predictors of shorter RFS. If this finding is validated in larger cohorts, testing of these genetic abnormalities may be used in routine practice to select patients with totally resected primary atypical meningioma at high risk of recurrence and who may require adjuvant post-surgical treatments. 

## Figures and Tables

**Table 1:** Clinical-pathological features and tumor mutational burden (TMB) of 22 atypical meningiomas.

Case	Sex	Age	Site	Histological Group	Sheeting	Necrosis	TMB (muts/Mb)	Simpson Grade	Recurrence	DFS (months)
1M	F	68	SB	Brain Invasion	no	Absent	12.68	1	no	54
2M	M	88	C	Brain Invasion	no	Absent	12.21	1	no	107
3M	F	83	C	Brain Invasion	no	Present	8.7	1	yes	9
4M	F	70	S	Brain Invasion	no	Absent	11.62	1	no	78
5M	M	69	S	Brain Invasion	no	Absent	2.19	1	yes	53
6M	F	67	C	Brain Invasion	no	Present	10.42	1	yes	68
7M	F	71	SB	Brain Invasion	no	Present	12.19	2	yes	54
8M	F	51	C	Brain Invasion	no	Present	6.56	1	yes	47
9M	F	36	C	High mitotic index	no	Present	9.32	1	no	62
10M	F	50	C	High mitotic index	yes	Present	6.61	1	no	61
11M	M	68	C	High mitotic index	yes	Present	10.25	3	yes	35
12M	F	44	C	High mitotic index	yes	Absent	11.89	1	no	47
13M	M	56	C	High mitotic index	no	Present	10.55	2	yes	68
14M	M	63	S	High mitotic index	yes	Present	9.76	3	yes	4
15M	M	60	C	High mitotic index	no	Absent	8.05	1	yes	8
16M	M	57	C	High mitotic index	yes	Present	11.47	1	yes	8
17M	F	54	S	Only minor criteria	no	Absent	8.87	3	no	89
18M	F	79	S	Only minor criteria	yes	Present	5.84	3	no	84
19M	M	76	C	Only minor criteria	no	Present	5.58	1	no	72
20M	F	71	S	Only minor criteria	yes	Present	7.36	2	yes	12
21M	M	77	S	Only minor criteria	no	Present	4.15	2	yes	5
22M	M	73	S	Only minor criteria	no	Present	7.3	2	yes	5

**Table 2:** Univariate analyses for recurrence-free survival (RFS) in 22 patients with atypical meningiomas.

Parameter	HR (95% CI)	*p*
Clinical-pathological risk score		
*low*	1	
*high*	3.5 (1.1–11.1)	0.03
Spontaneous necrosis		
*absent*	1	
*present*	2.7 (0.8–8.5)	0.087
18q deletion		
*no*	1	
*yes*	2.6 (0.7–7.6)	0.03
1p deletion		
*no*	1	
*yes*	3 (0.8–11.1)	0.111
10q deletion		
*no*	1	
*yes*	0.6 (0.1–3.7)	0.642
14q deletion		
*no*	1	
*yes*	1.9 (0.4–3.7)	0.346
22q deletion		
*no*	1	
*yes*	1.4 (0–4–4.8)	0.551
*NF2* mutation		
*no*	1	
*yes*	0.7 (0.2–2.3)	0.617
Genetic risk score		
*no*	1	
*yes*	5.3 (1.5–18.2)	0.008
CNL		
*low*	1	
*high*	2.3 (0.7–7.2)	0.145
TMB		
*low*	1	
*high*	0.6 (0.2–1.9)	0.423

HR: hazard ratio. CI: confidence interval. CNL: copy number losses. TMB: tumor mutational burden.

## Data Availability

The data presented in this study are available on request from the corresponding author. The data are not publicly available due to privacy issues.

## Supplementary Materials

The following are available online at https://www.mdpi.com/2072-6694/13/4/903/s1, Table S1: List of somatic and germline mutations identified in 14 of the 22 atypical meningiomas. Cases are sorted by ID sample and alphabetical order.
